# Supplementary material for: Quality of life among patients with the common chronic disease during COVID-19 pandemic in Northwest Ethiopia: A structural equation modelling
Source: PLoS One. 2022 Dec 6;17(12):e0278557. doi: 10.1371/journal.pone.0278557 (PMC9725128; doi:10.1371/journal.pone.0278557)
Supplement: S1 Table — (DOCX) [file pone.0278557.s001.docx]

S1 table: the measurement tool for quality of life adapted from WHO BREF

| q1 | How would you rate your quality of life? | 1. Very poor 2. poor 3. Neither poor nor good 4. Good 5. Very good |
| --- | --- | --- |
| q2 | How satisfied are you with your health? | 1. Very dissatisfied 2. Dissatisfied 3. Neither satisfied nor dissatisfied 4. Satisfied 5. Very satisfied |
| q3 | To what extent do you feel that physical pain prevents you from doing what you need to do? | 1. Not at all 2. A little 3. A moderate amount 4. Very much 5. An extreme amount |
| q4 | How much do you need any medical treatment to function in your daily life | 1. Not at all 2. A little 3. A moderate amount 4. Very much 5. Extremely |
| q5 | How much do you enjoy life? | 1. Not at all 2. A little 3. A moderate amount 4. Very much 5. Extremely |
| q6 | To what extent do you feel your life to be meaningful? | 1. Not at all 2. A little 3. A moderate amount 4. Very much 5. Extremely |
| q7 | How well are you able to concentrate? | 1. Not at all 2. A little 3. A moderate amount 4. Very much 5. Extremely |
| q8 | How safe do you feel in your daily life? | 1. Not at all 2. A little 3. A moderate amount 4. Very much 5. Extremely |
| q9 | How healthy is your physical environment? | 1. Not at all 2. A little 3. A moderate amount 4. Very much 5. Extremely |
| q10 | Do you have enough energy for everyday life? | 1. Not at all 2. A little 3. Moderately 4. Mostly 5. Completely |
| q11 | Are you able to accept your bodily appearance? | 1. Not at all 2. A little 3. Moderately 4. Mostly 5. Completely |
| q12 | Have you enough money to meet your needs? | 1. Not at all 2. A little 3. Moderately 4. Mostly 5. Completely |
| q13 | How available to you is the information that you need in your day-to-day life? | 1. Not at all 2. A little 3. Moderately 4. Mostly 5. Completely |
| q14 | To what extent do you have the opportunity for leisure activities? | 1. Not at all 2. A little 3. Moderately 4. Mostly 5. Completely |
| q15 | How well are you able to get around? | 1. Not at all 2. A little 3. Moderately 4. Mostly 5. Completely |
| q16 | How satisfied are you with your sleep? | 1. Very dissatisfied 2. Dissatisfied 3. Neither dissatisfied nor satisfied 4. Satisfied 5. Very satisfied |
| q17 | How satisfied are you with your ability to perform your daily living activities? | 1. Very dissatisfied 2. Dissatisfied 3. Neither dissatisfied nor satisfied 4. Satisfied 5. Very satisfied |
| q18 | How satisfied are you with your capacity for work? | 1. Very dissatisfied 2. Dissatisfied 3. Neither dissatisfied nor satisfied 4. Satisfied 5. Very satisfied |
| q19 | How satisfied are you with yourself? | 1. Very dissatisfied 2. Dissatisfied 3. Neither dissatisfied nor satisfied 4. Satisfied 5. Very satisfied |
| q20 | How satisfied are you with your personal relationships? | 1. Very dissatisfied 2. Dissatisfied 3. Neither dissatisfied nor satisfied 4. Satisfied 5. Very satisfied |
| q21 | How satisfied are you with your sex life? | 1. Very dissatisfied 2. Dissatisfied 3. Neither dissatisfied nor satisfied 4. Satisfied 5. Very satisfied |
| q22 | How satisfied are you with the support you get from your friends? | 1. Very dissatisfied 2. Dissatisfied 3. Neither dissatisfied nor satisfied 4. Satisfied 5. Very satisfied |
| q23 | How satisfied are you with the conditions of your living place? | 1. Very dissatisfied 2. Dissatisfied 3. Neither dissatisfied nor satisfied 4. Satisfied 5. Very satisfied |
| q24 | How satisfied are you with your access to health services? | 1. Very dissatisfied 2. Dissatisfied 3. Neither dissatisfied nor satisfied 4. Satisfied 5. Very satisfied |
| q25 | How satisfied are you with your mode of transportation? | 1. Very dissatisfied 2. Dissatisfied 3. Neither dissatisfied nor satisfied 4. Satisfied 5. Very satisfied |
| q26 | How often do you have negative feelings, such as blue mood, despair, anxiety, depression? | 1. Never 2. Seldom 3. Quite often 4. Very often 5. Always |

PHD=Physical Health Domain; PSHD=Psychological Health Domain; SRD=Social Relationship Domain and EHD=Environmental Health Domain
