# Supplementary material for: Quality of life among patients with the common chronic disease during COVID-19 pandemic in Northwest Ethiopia: A structural equation modelling
Source: PLoS One. 2022 Dec 6;17(12):e0278557. doi: 10.1371/journal.pone.0278557 (PMC9725128; doi:10.1371/journal.pone.0278557)
Supplement: Attachment — Submitted filename: Comments.docx [file pone.0278557.s002.docx]

Abstract and Introduction- Need to rephrase and construct for better sentences

Line 54-58 Need to check the sentences, the facts are contradicting with each other.

Line 96- suggest for ‘Facility based cross sectional study design’ to ‘A cross sectional study design’…

Methods- study setting

It is important to highlight during the study period, how many existing healthcare facilities are used for COVID-19, converted as hybrid healthcare facilities and healthcare facilities are not used for COVID-19. Not need to mention too detail about the burden of disease for COVID-19.

Can you explain about the semi-structured questionnaire used for your study?

What is the proportion of face-to-face interview before you switch to the electronic form?

Why were the validity and reliability not conducted for SRQ-20 tool and Morisky Medication Adherence Scale (MMAS-8)?

The chronic diseases are self-reported or confirmed with the medical records?

There are few typos error

How do you overcome information and recall bias in this study?

Should separate the categories able to read and write and the education level

Can the patient the differentiate the symptoms of the disease and complications of the disease?

How did you ask the common mental disorders for this study? (This is a diagnosis)

What was measured for ‘Kind of medication’?
