# Supplementary material for: Quality of life among patients with the common chronic disease during COVID-19 pandemic in Northwest Ethiopia: A structural equation modelling
Source: PLoS One. 2022 Dec 6;17(12):e0278557. doi: 10.1371/journal.pone.0278557 (PMC9725128; doi:10.1371/journal.pone.0278557)
Supplement: Attachment — Submitted filename: PLOS ONE_Reviewew new comments .docx [file pone.0278557.s004.docx]

| Quention | Reviewer Comments | Authors Response: | Reviewer New Comments |
| --- | --- | --- | --- |
| 6 | Why were the validity and reliability not conducted for SRQ-20 tool and Morisky Medication Adherence Scale (MMAS-8)? | Thank you dear reviewer for your concern, this tool is already widely used and validate for our country. | Please put the Cronbach Alpha value for SRQ-20 tool and Morisky Medication Adherence Scale (MMAS-8), and cited the papers for tools validation conducted in Ethiopia( as references) |
| 10 | Should separate the categories ‘able to read and write’ and the education level | Thank you for your concerns. First, we tried to fit a model that includes with educational level. However, the major limitation of SEM is that the determinants should be either continuous variable or binary if it is categorical. | For descriptive analysis (table 1), ‘Unable to read and write’ and ‘Able to read and write’ should be put under new variable e.g ‘Literacy status’. The 2 categories mentioned above are not compatible with the existing categories in the variable ‘education status’  For SEM analysis, you need to decide how to make the 3 categories for education status into binary. (same with marital status that have more than 2 categories) |
| 12 | How did you ask the common mental disorders for this study? (This is a diagnosis) | Authors Response: Thank you dear reviewer for your concern. We used self-reported  common mental disorders SRQ-20 tool and finally coding was conducted based on the  guideline of the tool. | The words ‘common mental disorder’ in the narrative of the results, tables, discussion and conclusion are not appropriate, because SRQ-20 tool is a screening tool for depression, anxiety symptoms and psychosomatic complaints.  Please change ‘common mental disorder’ with ‘mental health problem’. |
| 13 | What was measured for ‘Kind of medication’? | Thank you dear reviewer for your concern. We asked them how many kinds of drugs they took, and its unit was in numbers. | The use of ‘Kind of medication’ is not clear, unless you define it in the operational definition.    It is suggested the variable for ‘Kind of medication’ is change to ‘how many medication’. |
