# Supplementary material for: Quality of life among patients with the common chronic disease during COVID-19 pandemic in Northwest Ethiopia: A structural equation modelling
Source: PLoS One. 2022 Dec 6;17(12):e0278557. doi: 10.1371/journal.pone.0278557 (PMC9725128; doi:10.1371/journal.pone.0278557)
Supplement: Attachment — Submitted filename: Response to reviewers.docx [file pone.0278557.s005.docx]

August 2022

**Rebuttal letter**

Submission ID**:** PONE-D-21-23936R1

**Title:** Quality of life among patients with common chronic disease during COVID-19 Pandemic in Northwest Ethiopia; A Structural Equation Modelling

PLOS ONE

Tadesse Awoke Ayele, Habtewold Shibru, Malede Mequanent Sisay, Tesfahun Melese, Melkitu Fentie, Telake Azale, Tariku Belachew, Kegnie Shitu**,** and Tesfa Sewunet Alamneh*

**Dear Editor and reviewer,**

We would like to thank for your consideration and suggestion for the betterment our manuscript and make it more informative. We tried to amend the format of the manuscript according to the journal guidelines and address the questions raised by reviewer on the manuscript. Our point-by-point responses for each comment and questions are described in detail on the following pages. Further, the details of changes were shown by track changes in the supplementary document attached.

**Response to Reviewer**

1. Please put the Cronbach Alpha value for SRQ-20 tool and Morisky Medication Adherence Scale (MMAS-8) and cited the papers for tools validation conducted in Ethiopia (as references).

**Author’s response:** thank you dear reviewer and we appreciate your comment. We have included it.

2. For descriptive analysis (table 1), ‘Unable to read and write’ and ‘Able to read and write’ should be put under new variable e.g., ‘Literacy status’. The 2 categories mentioned above are not compatible with the existing categories in the variable ‘education status’

**Author’s response**: thank you dear reviewer and we appreciate your comment. We have changed to literacy status.

3. The words ‘common mental disorder’ in the narrative of the results, tables, discussion, and conclusion are not appropriate, because SRQ-20 tool is a screening tool for depression, anxiety symptoms and psychosomatic complaints.
   Please change ‘common mental disorder’ with ‘mental health problem’.

**Author’s response:** thank you dear reviewer and we appreciate your comment. We have updated it.

4. The use of ‘Kind of medication’ is not clear unless you define it in the operational definition. It is suggested the variable for ‘Kind of medication’ is change to ‘how many medication’.

**Author’s response:** thank you dear reviewer and we appreciate your comment. It was supposed to indicate how many medications were taken. As you mentioned, it is ambiguous, and we changed it to kind of medication according to make it clear.
