# Supplementary material for: Quality of life among patients with the common chronic disease during COVID-19 pandemic in Northwest Ethiopia: A structural equation modelling
Source: PLoS One. 2022 Dec 6;17(12):e0278557. doi: 10.1371/journal.pone.0278557 (PMC9725128; doi:10.1371/journal.pone.0278557)
Supplement: Attachment — Submitted filename: Plos One comments.docx [file pone.0278557.s006.docx]

1. For descriptive analysis (table 1), you only changed the ‘education status’ to ‘Literacy status’ and the categories remained the same and also their frequency and percentage.

(i) for the new variable ‘Literacy status’, the 2 categories under it are ‘Unable to read and write’ and ‘Able to read and write’. The total percentage of these 2 categories is 100%. Do not include the categories under variable ‘education status’ in this variable ‘ literacy status’.

(ii) Please maintain the variable ‘education status’. The 3 categories in this variable are Primary education, Secondary education and Diploma. Please ensure the total percentage of these 3 categories is 100%.

2. Please address my comment in the previous revision:

For SEM analysis, you need to decide how to make the 3 categories for education status into binary. (same with marital status that have more than 2 categories)
