# Supplementary material for: Quality of life among patients with the common chronic disease during COVID-19 pandemic in Northwest Ethiopia: A structural equation modelling
Source: PLoS One. 2022 Dec 6;17(12):e0278557. doi: 10.1371/journal.pone.0278557 (PMC9725128; doi:10.1371/journal.pone.0278557)
Supplement: Attachment — Submitted filename: Response to Reviwers.docx [file pone.0278557.s007.docx]

October 2022

**Rebuttal letter**

Submission ID**:** PONE-D-21-23936R1

**Title:** Quality of life among patients with common chronic disease during COVID-19 Pandemic in Northwest Ethiopia; A Structural Equation Modelling

PLOS ONE

Tadesse Awoke Ayele, Habtewold Shibru, Malede Mequanent Sisay, Tesfahun Melese, Melkitu Fentie, Telake Azale, Tariku Belachew, Kegnie Shitu**,** and Tesfa Sewunet Alamneh*

**Dear Editor and reviewer,**

We would like to thank for your consideration and suggestion for the betterment our manuscript and make it more informative. We undergone editing of the manuscript to address the questions raised by reviewer. Our point-by-point responses for both comments are described in detail on the following pages. Further, the details of changes were shown by track changes in the supplementary document attached.

**Response to Reviewer**

1. For descriptive analysis (table 1), you only changed the ‘education status’ to ‘Literacy

status’ and the categories remained the same and their frequency and percentage.

(i) for the new variable ‘Literacy status’, the 2 categories under it are ‘Unable to read and

write’ and ‘Able to read and write’. The total percentage of these 2 categories is 100%. Do

not include the categories under variable ‘education status’ in this variable ‘literacy status’.

(ii) Please maintain the variable ‘education status’. The 3 categories in this variable are

Primary education, Secondary education, and Diploma. Please ensure the total percentage of these 3 categories are 100%.

**Author’s response:** thank you dear reviewer and we appreciate your effort for the betterment of our work. We have tried to address your comment by including literacy status and educational level. As you mentioned it the literacy variable has 2 categories who can read and write and unable to read and write. Able to read and write individual were further classified to the three categories that you strike it. The frequency and detail editing are found in the revised version.

2. Please address my comment in the previous revision:

For SEM analysis, you need to decide how to make the 3 categories for education status into

binary. (Same with marital status that have more than 2 categories).

**Author’s response**: thank you dear reviewer and we appreciate your comment. After you suggestion we used to literacy status (which is binary in nature) instead of educational level after assuming all educated individual can read and write. We assumed that separated, widowed, and divorced individuals were married at certain time but it might cause loss of information since there might be heterogeneity among married, separated, widowed, and divorced individuals. Due this, we include the drawbacks of SEM which only consider either continuous or binary variable in the measurement model as a limitation in our work.
